# Clinicians’ attitudes toward standardized assessment and diagnosis within child and adolescent psychiatry

**DOI:** 10.1186/s13034-019-0269-0

**Published:** 2019-02-12

**Authors:** M. Danielson, A. Månsdotter, E. Fransson, S. Dalsgaard, J-O. Larsson

**Affiliations:** 1 Department of Women’s and Children’s Health, Karolinska Institutet, 171 77 Stockholm, Sweden; 2 Stockholm Child and Adolescent Psychiatry, Stockholm County Council, Box 17914, 118 95 Stockholm, Sweden; 3 Department of Public Health and Clinical Medicine, 901 87 Umeå, Sweden; 4 Centre for Health Equity Studies, Department of Public Health Sciences, Stockholm University, 106 91 Stockholm, Sweden; 5 Department of Microbiology, Tumor and Cell Biology, Karolinska Institutet, 171 77 Stockholm, Sweden; 6 The National Centre for Register-based Research (NCRR) and Centre for Integrated Register-based Research at AU (CIRRAU), Aarhus University, Fuglesangs Alle 4, 8210 Aarhus V, Denmark

**Keywords:** Standardized assessment, Implementation, Utility, Mental health service

## Abstract

**Background:**

There is a strong call for clinically useful standardized assessment tools in everyday child and adolescent psychiatric practice. The attitudes of clinicians have been raised as a key-facilitating factor when implementing new methods. An explorative study was conducted aimed to investigate the clinicians’ attitudes regarding standardized assessments and usefulness of diagnoses in treatment planning.

**Methods:**

411 mental health service personnel working with outpatient and inpatient assessment and treatment within the specialist child and adolescent mental health services, Stockholm County Council were asked to participate in the study, of which 345 (84%) agreed answer a questionnaire. The questionnaire included questions regarding Attitudes toward Standardized Assessment and Utility of Diagnosis. Descriptive analyses were performed and four subscales were compared with information from a similar study in US using the same instruments. The demographic and professional characteristics (age, working years, gender, education, profession, management position, involvement in assessment, level of service) in terms of prediction of attitudes were studied by univariate and multivariate linear regressions.

**Results:**

Overall, the clinicians had quite positive attitudes and were more positive compared to a similar study conducted in the US earlier. There were differences in attitudes due to several characteristics but the only characteristic predicting all subscales was type of profession (counselor, nurse, psychiatrist, psychologist, other), with counselors being less positive than other groups.

**Conclusion:**

The overall positive attitudes toward standard assessment are of importance in the development of evidence-based practice and our study implies that clinicians in general value and are willing to use standardized assessment. Nevertheless, there are specific issues such as adequate training and available translated assessment instrument that need to be addressed. When implementing new methods in practice, there are general as well as specific resistances that need to be overcome. Studies in different cultural settings are of importance to further extend the knowledge of what is general and what is specific barriers.

## Introduction

Over recent decades the field of child and adolescent mental health care has changed and the demand for obtaining structured, systematic and valid information of diagnosis and treatments has increased, in order to prioritize and plan organization of mental health services [1, 2]. Parallel to these changes, the health care systems have been influenced by the evidence-based movement highlighting the importance of using scientific findings in decision-making [3]. An overarching concept in this movement is evidence-based practice (EBP), characterized as a systematic approach, integrating best research evidence and standardized data, with clinical expertise, while respecting patient preferences [4–6]. Although many different evidence-based initiatives have been undertaken in within the field of child and adolescent psychiatry, EBP has so far only been implemented in slow pace within this specialty [7, 8].

Appropriate diagnosis is essential for providing good medical and psychological treatments and for psychoeducation, i.e. helping patients and their families to recognize and understand symptoms [9–11]. Valid and accurate diagnoses are also stipulated in treatment protocols and are prerequisites for planning accurate interventions [10].

Making a diagnosis requires thorough assessment of medical history, symptoms, and function. Yet, traditionally the diagnostic assessment by clinicians has been more or less unstructured, capturing some but not all of the diagnostic criteria described in the disease classifications [12, 13]. A recent study within adult psychiatry showed that clinicians do not collect sufficient information in order to establish a correct diagnosis [14]. Furthermore, the traditional diagnostic process and the information obtained by this has consequently been subject to considerable variation [15].

The importance of standardized diagnostic interviews in child and adolescent psychiatry practice has been highlighted in several studies [12, 13, 16, 17], as well as within the field of clinical psychology [18]. Standardized diagnostic interviews are assumed to save time and speed up the assessment process by facilitating and clarifying the diagnostic process, systematically detect comorbidity, obtain a reliable diagnosis and prepare treatment in a more solid manner [10, 19]. Less use of structured interviews has been related to underestimation of patient acceptance and mistaken assumptions of patients’ feelings [20].

Despite the importance of assessment, most attention has been given to Evidence-Based Treatments (EBT) and not that much to assessments in the EBP literature [2, 5, 21]. However, during recent years, the concept of Evidence-Based Assessment (EBA) has been launched as a part of EBP. Mash and Hunsley [22] propose that standardized assessments (SA) are not restricted to standardized interviews and could be conducted for other purposes than determining diagnosis, such as prognosis and predictions, treatment planning and monitoring. Similarly, Christon et al. [23] have proposed how EBA could be part of EBP in the treatment process.

EBA represents a strong call for valid and clinically useful assessment tools in everyday child and adolescent psychiatric practice; both for strengthening the diagnostic process and enabling ongoing progress monitoring [24, 25]. Nevertheless, a survey among 1.927 psychiatrists and psychotherapists in Switzerland revealed that on average only 15% of the patients were assessed using standardized assessment tools [20]. Further, Garland, Kruse and Aarons [1] found that standardized measures or scales were even less frequently used within child and adolescent psychiatric settings; 92% of child psychiatrists indicated they had never used the scores from standardized measures in their clinical practice. An inventory conducted in Sweden found that 39% of all psychiatric units used standardized assessment tools in the diagnostic process but not frequently and only 12% did so on regularly basis [26].

A key facilitating factor for the general success of implementing methods or innovations is whether clinicians find the procedures relevant [27]. Earlier studies have shown that clinicians’ incitement for diagnosing is often external e.g. billing purposes, rather than usefulness, which reduces investment in the assessment process [28, 29]. Concerns for using SA in the assessment process have also been highlighted and the arguments against using SA include that they are time consuming, that structured interviews will disturb the therapeutic relationship and that clinical judgments are more sufficient and useful [25, 20]. In parallel, a review of therapist-level resistance to EBP showed that psychotherapists believe that they can objectively and without bias perceive the patients’ problem and treatment outcome [30]. Harvey and Gumport [31] have identified obstacles against EBT in general and call for more studies of therapists’ beliefs and preferences among a broader range of mental health professionals. The same call could probably be made about EBA since there are even fewer studies conducted.

Implementation of new clinical procedures is strongly influenced by clinicians’ attitudes. However, there is not yet enough knowledge about obstacles towards the use of standardized tools in diagnostic assessment processes. Large scale studies of child and adolescent mental health providers from various disciplines and in different countries is needed to inform specific efforts to encourage clinicians to use standardized tools systematically and thereby to more evidence-based assessments.

This study is an exploratory study and aims to investigate clinician´s attitudes towards standardized assessments and usefulness of diagnosis, and the research questions are:

- What are the attitudes of clinicians in secondary mental health care in Stockholm, Sweden towards standardized assessment and the usefulness of diagnosis in treatment planning and how do they differ from an US population?
- Do Swedish clinicians’ attitudes differ between groups due to demographic and profession?

## Method

### Participants and setting

In Sweden, the child and adolescent mental health services are divided into two parts: (1) the primary mental health care (general physicians and psychologists, not licensed as specialists in child and adolescent mental disorders) and (2) the specialized mental health care (licensed specialists, i.e. psychiatrics/child psychiatrists and psychologist specialized in mental disorders working in multidisciplinary teams, together with nurses, counselors and others). The present study was conducted within the latter. The participants were mental health care personnel working with outpatient and inpatient assessment and treatment within the non-private specialist child and adolescent mental health care services in Stockholm County Council (CAMHS Stockholm). Each year, approximately 22,000 children and adolescents receive treatment for a mental disorder in one of the six departments in CAMHS Stockholm. This equals nearly 6% of the population under 18 years of age in the catchment area. CAMHS Stockholm consists of 12 outpatient clinics, four intermediate care units mainly working with patients in their home or other environments and one inpatient clinic. All 411 mental health service personnel working with assessment and treatment were asked to participate in the study, of which 345 (84%) volunteered to participate. CAMHS Stockholm also includes seven outpatient clinics specialized in treating e.g. sexual abuse, selfharm, domestic violence, immigrants with mental health problems, and to which patients are referred after initial assessment in the general clinics. Hence, clinicians in the specialized clinics were not included in this survey.

There were mainly female participants (78%) and the average age was 47.2 years (median 48). The participants had worked within the children and adolescent mental health services for an average of 10.3 years (median 7). The participants were psychologists (49%), counselors with a degree in social work and psychotherapy, (22%), medical doctors/psychiatrists (10%), nurses (9%) and other occupational background like mental health keepers, pedagogues etc. with therapeutic training (8%). The majority of participants (90%) had more than 3.5 years of education from university. All the clinical staff working at the CAMHS Stockholm are involved in interdisciplinary assessments in the beginning of a new patient contact, but not all conduct in-depth assessments involving psychological, medical and/or observational tests. The characteristics of the participants are further presented in Table 1.

**Table 1:** Distribution of participants’ demographic and professional characteristics (n = 345)

Demographic characteristics	
Age (years)	
Mean (SD)	47.2 (11.8)
Unknown/missing data	0.6%
Gender	
Female	78.3%
Male	19.7%
Unknown/missing data	2.0%
Professional characteristics	
Working years within CAMHS	
Mean (SD)	10.3 (9.6)
Unknown/missing data	3.5%
Highest degree	
PhD	3.8%
University more than 3.5 years	85.5%
University less than 3.5 years	7.8%
Other higher education	1.4%
Unknown/missing data	1.4%
Profession	
Counsellor	22.0%
Nurse	8.7%
Psychiatrist/MD	10.1%
Psychologist	49.3%
Other	7.8%
Unknown/missing data	2.0%
Management position	
Yes	5.5%
No	90.7%
Unknown/missing data	3.8%
Conduct in-depth assessments	
Yes	81.4%
No	17.1%
Unknown/missing data	1.4%
Level of service	
Outpatient	73.0%
Intermediate	17.4%
Inpatient	9.0%
Unknown/missing data	0.6%

### Procedure

In each of the participating clinics, the clinic manager distributed a questionnaire either during staff meetings or individually distributed in internal mailboxes. During the period when the survey was conducted, 461 were employed, although 50 of them did not receive the questionnaire due to different circumstances, i.e. long sick leave, educational leave or vacation etc. If the clinicians volunteered to participate they completed the questionnaire individually and anonymously and returned the surveys directly to the researchers, using sealed envelopes.

### Measures

The questionnaire included questions regarding demographic and professional characteristics (independent variables), the measurement Attitudes toward Standardized Assessment (ASA) consisting of four subscales and the Utility of Diagnosis scale (dependent variables) developed in earlier studies [24, 25]. The scales were translated in collaboration with researchers in Norway and Denmark, and back-translated. One of the original developers of the questionnaire, Dr. Jensen-Doss audited the back-translation to secure correct meaning and approved the final translated Swedish version.

#### Demographic and professional characteristics

The demographic and professional characteristics included age, number of years working within CAMHS, gender, highest educational degree (categorized as PhD; university more than 3.5 years; university less than 3.5 years/other higher education), profession (categorized as counselor; nurse; psychiatrist/MD including those on specialist training; psychologist; other), management position (categorized as unit manager or co-manager of the clinic or not), degree of involvement in assessments (conducting in-depth diagnostic examinations or not) and level of service (outpatient; intermediate; inpatient). In this context, the clinicians’ psychotherapeutic training was of interest, as CBT (cognitive behavioral therapy) has a long tradition of using assessments [32]. However, since most participants had a broad therapeutic educational training, indicating an eclectic approach, this factor could not be explored in the analysis.

#### Attitudes toward standardized assessment and usefulness of diagnosis

The ASA questionnaire was originally developed to assess clinicians’ attitudes toward SA in three different areas, each measured by a subscale [25]. In total, ASA consists of 22 items, all rated on a 5-point Likert scale from 1 (strongly disagree) to 5 (strongly agree). The questionnaire measures both positive and negative attitudes towards standardized assessments. Hence, in order to receive a universal ranking of the scale direction, the negative ranking scores were re-coded to correspond to the positive ranking scores. For each subscale, the average ranking of included items were calculated.

The ASA subscale, *Benefit over Clinical Judgment* assesses to which extent standardized tools can improve the assessment information compared to relying on clinical judgments alone. The scale consists of five items and with the internal consistency α = .75 in the present study. The subscale, *Practicality* assesses clinician opinions of the feasibility in practice, and consists of 10 items with the internal consistency α = .60 in the present study. The subscale, *Psychometric Quality* assesses clinicians’ beliefs concerning reliability and validity of standardized measures and how much they value these psychometric properties, and consists of 7 items with the internal consistency α = .69.

Separate from ASA, *The Utility of Diagnosis* scale assesses clinicians' opinions regarding the usefulness of diagnosis in their clinical work (e.g. “Making a diagnosis is more important for obtaining services or benefits than for planning of treatment”) since it could be of importance for the willingness to invest in the assessment process. The subscale was developed by the same founders as ASA [24] and consists of five items, also rated on a 5-point Likert scale from 1 (strongly disagree) to 5 (strongly agree), but with somewhat lower internal consistency (α = .45) than the subscales included in ASA. When single items were excluded from the scale in further reliability analysis, the internal consistency improved somewhat, α = .50, and when keeping only three items, it improved additionally (α = .54). However, our judgment was that these improvements were not large enough to motive change of the scale, and we decided to keep all items of the original scale.

### Data analysis

Prior to analysis we examined normal distribution of continuous independent and dependent variables using test of skewness and kurtosis in which values between − 2 and 2 are considered acceptable, according to Almquist, Ashir and Brannstroem [33]. The two independent variables, age and working years, were somewhat skewed, whereas the four dependent variables, the attitude subscales, fulfilled criteria for normality.

In order to explore the first research question on clinicians attitudes regarding standardized assessment and diagnosis and how they differ from an US population, descriptive statistics were performed and the four subscales were compared with information from a similar study in US [24, 25] using an immediate form of two-sample *t* test, *ttesti* in Stata [34].

In preparation to answer the second research question on differences between clinicians due to characteristics, descriptives of the four subscale (means and standard deviations) were first calculated by categories of each demographic and professional characteristic and then tested in an ANOVA and Post Hoc analysis. The two continuous variables, age and number of working years within secondary mental health services, were dichotomized at the median. As a result of the ANOVA and Post Hoc analysis, three independent variables were changed. Highest educational degree was dichotomized by merging “Doctorial” and “University more than 3.5 years” and by merging “University less than 3.5 years” and “Other higher education”. Second Level of service was dichotomized by merging “Outpatient” and Intermediate” into one category, and keeping “Inpatient” as the other category. Third, profession categories “Nurse” and “Other” were merged into one category.

In order to answer the second research question whether clinicians’ attitudes in Sweden differ between groups due to demographic and professional characteristics and to what degree the same characteristics predict the attitudes univariate and multivariate linear regressions were conducted. In the regression analyses, the continuous data for age and working years within secondary children and adolescent mental health services were used [35].

Since age and working years within secondary children and adolescent mental health services were strongly correlated, r (331) = .69, p < .000, we considered excluding one of them from the multivariate analysis. However, this did not change the explained variance, and hence, both variables were kept in the model, making it possible to explore the strengths of prediction for both of them. In order to compare profession categories, the multivariate regressions were conducted for each category compared to another, one at a time (Psychiatrist/MD vs psychologist; Psychiatrist/MD vs councellor; Psychiatrist/MD vs nurses/other; Psychologist vs councellor; Psychologist vs nurses/other; Councellor vs nurses/other).

Missing data were examined with Post Hoc analysis of the variance for each dependent variable value. This showed that participants with missing data did not differ from the others. The number of missing data on each characteristic is presented in Table 1 and since the overall rate of missing data was low, 5% or fewer, decision was made to use listwise deletion.

Decision was made to choose significant level 95% in all analyses and utilize the alphas of .05. Cohen’s definitions of effect sizes [36] were used to describe the subscale differences between two samples t-tests, d values of .20, .50 and .80 were interpreted as small, medium and large effect, and the strengths of the regression coefficients, R^2^ values of .02, .13 and .26 were interpreted as small, medium and large effect sizes [37].

## Results

### Clinicians’ attitudes to standardized assessments and diagnoses

The clinicians’ attitudes to standardized assessments and diagnostic interviews and to the usefulness of diagnosis in clinical work are presented in Table 2.

**Table 2:** Descriptive statistics of subscales and items for Attitudes toward Standardized Assessment and Utility of diagnosis in CAP Stockholm (point scales, means, standard variation, N) and comparison to US (mean, standard deviation, N)

Subscales and items within each scale	CAP Stockholm						US^a^	Diff between CAP Stockholm—US^c^
	Strongly disagree %	Disagree %	Neutral %	Agree %	Strongly agree %	M (SD) N	M (SD) N	
Benefit over clinical judgment						3.14 (0.65) 338	2.95 (0.68) 1439	***
Using clinical judgment to diagnose children is superior to using standardized assessment measures^b^	4.5	25.2	47.4	18.0	4.8	2.93 (0.90) 333	3.16 (0.96) 1439	***
Standardized measures don’t capture what’s really going on with children and their families^b^	5.3	34.7	40.7	17.2	2.1	2.76 (0.87) 337	3.11 (0.95) 1439	***
Clinical problems are too complex to be captured by a standardized measure^b^	5.4	31.8	29.5	27.1	6.3	2.97 (1.03) 336	3.02 (0.98) 1439	ns
Standardized measures provide more useful information than other assessments like informal interviews or observations	7.5	34.4	42.2	13.2	2.7	2.69 (0.90) 334	2.5 (0.82) 1439	***
Standardized measures don’t tell me anything I can’t learn from just talking to children and their families^b^	15.2	53.3	19.0	9.8	2.7	2.32 (0.94) 336	2.47 (1.06) 1439	*
Practicality						3.19 (0.44) 338	3.19 (0.56) 1404	ns
Standardized measures can efficiently gather information from multiple individuals (e.g. children, parents, teachers)	0.6	3.0	11.9	57.3	27.3	4.08 (0.75) 337	3.91 (0.79) 1404	***
Standardized assessments are readily available in the language my children and their families speak	21.4	29.4	41.5	6.5	1.2	2.38 (0.93) 337	3.34 (1.12) 1404	***
There are few standardized measures valid for ethnic minority children and their families^b^	1.2	3.9	37.9	34.8	22.1	3.73 (0.89) 330	3.32 (0.82) 1404	***
I have adequate training in the use of standardized measures	3.3	13.9	18.6	38.2	26.0	3.70 (1.10) 338	3.25 (1.24) 1404	***
Standardized diagnostic interviews interfere with establishing rapport during an intake^b^	15.4	28.8	27.0	18.4	10.4	2.80 (1.20) 337	3.04 (1.09) 1404	***
Standardized measures take too long to administer and score^b^	7.4	31.0	35.1	21.4	5.1	2.90 (1.00) 336	2.99 (1.07) 1404	ns
Standardized symptom checklists are too difficult for many children and their families to read or understand^b^	3.3	27.0	44.8	22.6	2.4	2.94 (0.85) 337	2. 72 (0.92) 1404	***
Copyrighted standardized measures are affordable for use in practice	2.7	5.4	74.4	12.3	5.1	3.12 (0.69) 332	2.71 (0.99) 1404	***
Completing a standardized measure is too much of a burden for children and their families^b^	13.9	45.7	33.8	6.2	0.3	2.33 (0.80) 337	2.69 (0.93) 1404	***
The information I receive from standardized measures isn’t worth the time I spend administering, scoring and interpreting the results^b^	10.4	43.0	32.9	11.0	2.7	2.53 (0.92) 337	2.58 (1.08) 1404	ns
Psychometric quality						3.81 (0.49) 340	3.78 (0.50) 1428	ns
Clinicians should use assessments with demonstrated reliability and validity	0.9	1.5	12.2	42.7	42.7	4.25 (0.79) 337	4.20 (0.83) 1428	ns
Standardized measures help with accurate diagnosis	1.2	2.7	16.9	48.8	30.5	4.05 (0.83) 338	3.91 (0.77) 1428	**
Standardized measures help detect diagnostic comorbidity (presence of multiple diagnoses)	0.3	2.4	24.8	53.7	18.8	3.90 (0.74) 335	3.67 (0.72) 1428	***
Standardized measures help with differential diagnosis (deciding between 2 diagnoses)	0.6	5.4	29.0	49.6	15.5	3.74 (0.80) 335	3.64 (0.78) 1428	*
Standardized measures overdiagnose psychopathology^b^	6.0	25.7	47.2	19.4	1.8	2.85 (0.86) 335	2.84 (0.89) 1428	ns
Most standardized measures aren’t helpful because they don’t map on to DSM diagnostic criteria^b^	14.3	38.7	42.9	3.6	0.6	2.38 (0.79) 336	2.45 (0.84) 1428	ns
It is not necessary for assessment measures to be standardized in research studies^b^	37.7	37.4	16.9	5.6	2.4	1.98 (0.99) 337	1.68 (0.84) 1428	***
Utility of diagnosis						3.60 (0.55) 330	3.15 (0.71) 1634	***
Accurate diagnosis is an important part of my treatment planning.	0.3	0.0	9.9	39.2	50.6	4.40 (0.69) 330	3.96 (0.93) 1634	***
Most children and families come to work on problems of daily life rather than being diagnosed^b^	1.2	7.6	26.8	48.2	16.2	3.70 (0.86) 328	3.72 (1.07) 1634	ns
It is sometimes necessary to give a diagnosis that is not clinically indicated to qualify for services^b^	28.9	31.3	23.0	12.1	4.7	2.31 (1.15) 327	2.89 (1.22) 1634	***
Making a diagnosis is more important for obtaining services or benefits than for planning of treatment^b^	20.9	31.8	31.5	12.9	2.9	2.46 (1.05) 328	2.88 (1.23) 1634	***
It is sometimes necessary to make a less serious diagnosis than clinically indicated to avoid stigma attached to serious diagnoses^b^	41.5	33.5	16.8	7.4	0.9	1.94 (0.98) 328	2.72 (1.14) 1634	***

*** p < .001 ** p < .01 * p < .05

^a^Jensen-Doss and Hewley [24, 25]

^b^Item was reverse scored before included in the scale score

^c^Using an immediate form of two-sample t-test, ttesti in Stata

First, the clinicians in Stockholm CAP were most positive concerning *Psychometric Quality* (M = 3.81 *CI* 3.76; 3.87). According to the confidence intervals they were less positive to *Utility of Diagnosis* (M = 3.60 *CI* 3.54; 3.66) and even less positive to the feasibility in Practice (M = 3.19 *CI* 3.13; 3.23) and *Benefit over Clinical Judgement* (M = 3.14 *CI* 3.07; 3.21). In comparison to US, clinicians in the Swedish setting were more positive concerning *Benefit over Clinical Judgment* (p < .001; Cohens *d *= .28) and to the *Utility of Diagnosis* (p < .001; Cohens *d *= .71) corresponding to small and medium effect sizes, respectively. No statistically significant differences between countries were found in attitudes concerning *Psychometric quality* (p > .05; Cohens *d *= .06) and *Practicality* (p > .05; Cohens *d *= .00).

Table 2 also present results at the single item level and here the Swedish clinicians were most negative concerning the availability of standardized measurements in other languages valid for ethnic minorities.

### Differences in attitudes by groups of demographic and professional characteristics

The descriptive results for each attitude subscale are presented by groups of demographic and professional characteristics in Table 3.

**Table 3:** Means (M) and standard deviations (SD) for clinicians’ attitudes to standardized assessment and utility of diagnosis by groups of demographic and professional characteristics

Characteristics	Benefit over clinical judgement		Practicality		Psychometric quality		Utility of diagnosis	
	*M *	*SD*	*M *	*SD*	*M *	*SD*	*M *	*SD*
Demographic characteristics								
Age (years)								
< 48	3.23	0.63	3.24	0.47	3.93	0.50	3.61	0.51
> 47	3.06	0.67	3.13	0.40	3.70	0.46	3.60	0.59
Gender								
Female	3.09	0.66	3.17	0.44	3.79	0.49	3.60	0.55
Male	3.34	0.57	3.26	0.44	3.87	0.49	3.65	0.51
Professional characteristics								
Working years within CAMHS								
< 8	3.22	0.63	3.21	0.47	3.88	0.50	3.59	0.51
> 7	3.08	0.66	3.17	0.41	3.76	0.48	3.62	0.60
Highest educational degree								
PhD	3.78	0.76	3.37	0.40	4.14	0.53	3.55	0.37
University more than 3.5	3.12	0.64	3.18	0.44	3.83	0.48	3.63	0.55
University less than 3.5	3.10	0.68	3.17	0.43	3.59	0.45	3.55	0.53
Other higher education	3.05	0.25	3.08	0.50	3.50	0.41	2.75	0.25
Profession								
Counsellor	2.80	0.63	3.03	0.45	3.60	0.38	3.53	0.56
Nurse	3.14	0.47	3.15	0.31	3.61	0.50	3.50	0.48
Psychiatrist/MD	3.49	0.63	3.41	0.40	4.03	0.51	3.85	0.65
Psychologist	3.20	0.64	3.21	0.44	3.93	0.47	3.65	0.49
Other	3.22	0.61	3.18	0.43	3.61	0.51	3.30	0.66
Management position								
Yes	3.21	0.62	3.28	0.51	3.85	0.48	3.63	0.79
No	3.13	0.65	3.18	0.43	3.82	0.49	3.61	0.53
Conduct indepth assessments								
Yes	3.15	0.68	3.20	0.45	3.86	0.48	3.62	0.55
No	3.06	0.51	3.13	0.39	3.57	0.45	3.56	0.52
Level of service								
Outpatient	3.12	0.66	3.17	0.44	3.83	0.49	3.64	0.54
Intermediate	3.09	0.61	3.15	0.41	3.71	0.49	3.47	0.53
Inpatient	3.43	0.52	3.35	0.43	3.91	0.50	3.61	0.64

The demographic and professional characteristics in terms of prediction of attitudes were studied by univariate and multivariate linear regressions and are presented in Table 4.

**Table 4:** Demographic and professional characteristics as predictors of clinician attitudes by four subscales; univariate (one independent variable) and multivariate (controls for all other independent variables) linear regressions

	Benefit over Clinical Judgement			Practicality			Psychometric Quality			Utility of Diagnosis		
	Univariate		Multivariate	Univariate		Multivariate	Univariate		Multivariate	Univariate		Multivariate
	*β*	R^2^	*β*	*β*	R^2^	*β*	*β*	R^2^	*β*	*β*	R^2^	*β*
Age (years)	− .139*	.019	− .038	− .086	.007	− .079	− .250***	.062	− .071	− .064	.004	− .043
Gender												
Female (1) vs male (0)	− .152**	.023	− .149**	− .088	.008	− .070	− .063	.004	− .055	− .040	.002	− .027
Working years within CAMHS	− .155**	.024	− .161*	− .008	.000	.025	− .186***	.035	− .110	.000	.000	.042
Highest educational degree												
Less than 3.5 years university (1) vs more than 3.5 years university (0)	− .022	.000	.006	− .024	.001	.009	− .153**	.023	− .014	− .095	.009	− .055
Profession		.099			.059			.123			.052	
Psychiatrist/MD (1) vs psychologist (0)	.225*		.175	.225*		.171	.104		.069	.187*		.178
Psychiatrist/MD (1) vs councellor (0)	.450***		.468***	.366***		.341***	.368***		.307***	.242***		.236*
Psychiatrist/MD (1) vs nurses/other (0)	.174*		.164	.206**		.142	.326***		.212*	.310***		.264
Psychologist (1) vs councellor (0)	.260***		.320	.177**		.197**	.281***		.249***	.085		.087
Psychologist (1) vs nurses/other (0)	.010		.042	.042		.023	.250***		.164*	.171**		.138
Councellor (1) vs nurses/other (0)	− .215***		− .223**	− .111		− .140	.005		− .044	.096		.064
Management position												
No (1) vs yes (0)	− .029	.001	− .109	− .051	.003	− .076	− .014	.000	− .066	− .010	.000	− .022
Conduct indepth assessments												
Yes (1) vs no (0)	.053	.003	− .042	.059	.003	− .012	.221***	.049	.079	.041	.002	− .059
Level of service												
Inpatient (1) vs Outpatient/intermediate (0)	.138*	.019	.114*	.121*	.015	.112	.063	.004	.074	.002	.000	.042

*** p < .001 ** p < .01 * p < .05

Profession alone explained 9.9% of the variance in the subscale *Benefit over Clinical Judgement* (*F(3327) *=* 11.96, p *< *.001*), a small effect size. Also gender (*F(1329) *=* 7.73, p *< *.010)* and working year (*F(1326) *=* 8.00, p *< *.001)* had small effect sizes. Entering all predictors into a multivariable regression analysis, they all together explained 17.3% of the variance (*F(10,281) *=* 7.08, p *< *.001)*, a medium effect size. Most predictors from the univariate analysis remained significant, except for age and the differences between psychiatrist and the other professions (Table 4).

According to univariate analysis profession explained 5.9% of the variance in *Practicality* scale (*F(3327) *=* 6.81, p *< *.001)*, a small effect size. Entering all predictors into a multivariate regression analysis they all together explained 6.2% of the variance (*F(10,281) *=* 2.94, p *< *.001)*, a small effect size.

Profession alone explained 12.3% of the variance in the subscale *Psychometric Quality* (*F(3329) *=* 15.40, p *< *.001),* medium effect size. Clinicians age explained 6.2% of the variance *(F(1336) *=* 22.33, p *< *.001),* working years explained 3.5% of the variance *(F(1328) *=* 11.74, p *< *.001)* and whether they conduct in-depth assessments or not explained 4.9% (*F(1328) *=* 11.74, p *< *.001);* all predictors had small effect sizes. According to the multivariate regression analysis all predictors together, explained 13.0% of the variance *(F(10,283) *=* 5.36, p *< *.001)*; a medium effect size, with only profession still being statistically significant.

The only statistically significant predictor of the subscale *Utility of Diagnosis* in the univariate regression was profession, which explained 5.2% of the variance *(F(3331) *=* 6.02, p *< *.001).* Entering all predictors into a multivariate regression analysis they all together explained only 1.8% of the variance in the model *Utility of Diagnosis* (*F(10,285) *=* 1.55, p *= *.122);* none effect size. Only one predictor remained statistically significant, psychiatrists were more positive than counselors.

## Discussion

This study aims to investigate attitudes of clinicians in specialist child and adolescent mental health care in Stockholm, Sweden towards standardized assessment and the usefulness of diagnosis in treatment planning and how do they differ from an US population.

The main finding from the present study is that clinicians in CAMHS Stockholm overall had quite positive attitudes towards the use of standardized assessment tools and found diagnoses useful. The attitudes were more positive compared to a similar previous study conducted in the US [24, 25]. The only characteristic that predicted attitudes, in all subscales, was profession.

Participants were most positive towards the psychometric quality of standardized assessments and the utility of diagnoses. They were somewhat less positive to the usefulness in practice and the use of standardized assessment when compared to clinical judgment. The patterns in attitudes across subscale were similar to those found in the US study [24, 25]. Exceptions from this included that clinicians in the present study seemed to be more positive towards the utility of diagnosis in treatment, compared to those in the US study. This is interesting since the health care systems in these two countries are somewhat different. Clinicians in Sweden were also more likely than their colleagues in US, to report that standardized tools improve the assessment information more than simply relying only on clinical judgments.

Our study also aimed to explore whether clinicians’ attitudes differ due to demographic and professional characteristics. The only characteristic that was found to predict attitude across all subscales was profession, with counselors being less positive than the other groups. Also, clinicians with fewer years of working within CAMHS, seemed to be more positive than those with longer experience, but this relationship was not sustained when controlling for all the other variables. The characteristics predicting the explained variance differed somewhat from those in the earlier mentioned US study by Jensen-Doss and Hewley [24, 25]. Even though profession seemed to be the most important predictor in both populations, it was not always the same professional groups that were most positive. This may be explained by cultural differences between countries, i.e. how the mental health services are organized, but also by differences in the duties/tasks, educational backgrounds and social status of the profession [38].

One previously identified barrier against EBP in general is the belief that it could have negative impact on the therapeutic relationship [39]. The clinicians in our study are not quite that pessimistic, which is positive from an implementation point of view [40]. However, the Swedish clinicians believe that SA do not offer additional information they cannot obtain from informal interviews or from just talking with the children and their parents. This finding is in line with other research; a review of therapist-level resistance to EBP showed that psychotherapists believe that they can objectively and without bias perceive the patients’ problem and treatment outcome [30] a belief not likely to be true [14, 20]. As mentioned earlier, our study indicates that less experienced clinicians in terms of working years within CAP were more positive than experienced clinicians, this was specifically to the use of SA over clinical judgements. This could be explained by the fact that less experienced appreciate more support in the diagnostic process, but also, as Nakamura, Higa-McMilla, Okamura and Shimabukuro suggest, by a more recent education at university, influenced more by EBP [41].

The results from our study raise practical issues that need to be considered. First few clinicians in our study, and even fewer than in the US study, agreed that assessment instruments in languages that their clients speak are readily available. Addressing the language issue is crucial, since assessment strategies need to be not just scientifically sound, but also culturally sensitive and clinically relevant as well [42]. As the patient group in mental health services has changed over the last decades in Sweden, with increasing proportions of children and adolescents originating from countries other than Sweden, it is important to consider availability of the instruments for the most common languages, when implementing EBA in clinical practices.

Second, about one-third of the clinicians reported that they did not have adequate training in using structured assessment tools, which implies a need for more education and practice in this area. According to several implementation strategy theories, e.g. Rogers theory Diffusion of innovations [43] and research within EBP [44], providers must not only have favorable attitudes towards it, they also need to have knowledge about the new technique, before successfully adopted into clinical practice. In a recent study of clinicians training in cognitive behavioral therapy with a strong focus on SA tools, the researchers used the ASA questionnaire to investigate the change in attitudes and use of SA before and after training and found that the clinicians developed a more positive attitude towards psychometric quality and feasibility of SA in clinical practices with training [45]. The actual use of SA also increased during training, but declined somewhat after the training was ended. This is in line with another study showing that training has an positive impact on attitudes and self-efficacy regarding using SA [46]. To continue increased use of SA, a learning environment is probably needed.

Finally, even if the practical issues are solved, successful implementation of EBA requires a competent and skilful organizational culture with commitment among the mental health service personnel [7]. As organizations and technologies change rapidly, solutions must be able to handle complex clinical situations and also be flexible. The arena where the patients (especially the young ones) and professionals meet will be somewhat different in the future [47]. This will also be the future for SA. Although the development of technological solutions has exploded during last decades, it is important that this trend continues in collaboration between clinicians and patients [48] as well as between practitioners and researchers [49].

Exchange of scientific and applied knowledge to meet these challenges within nations and between societies are therefore of importance. Whether EBA will be implemented in child mental health services or not in the future, does not only depend on clinicians’ attitude, knowledge, ability and motivation. The importance of organizational factors and resources have also been highlighted [50]. Generally, the motives for using SA must be clear and supported by suitable systems of the mental health service as well as science. When implementing EBP in the future an integrating approach is needed [23] where both EBA and EBT are of importance since they bridge the gap between science and community services [2].

### Strengths and limitations in our study

The present study is an investigation within only one of many Swedish Counties. However, almost a quarter of the Swedish population lives within the Stockholm County and the CAMHS Stockholm serves more than 80% of the population in this age group in the catchment area. In addition, the high response rate in our study and our respondent coverage of all professions within secondary children and adolescent mental health services, increase the generalizability of our findings.

Our study did not include data from the US and therefore it is important to be careful when drawing conclusions about the differences between the two national settings, Sweden and the US. The findings could, apart from pointing at possible cultural differences, also to some extent, be due to differences in the samples and applied methodology in obtaining and analyzing data.

Profession was the main characteristic statistically significantly associated with the results from all subscales and except for cultural professional differences, the size of the professional groups differed. Counselors were quite narrowly defined as a group in the Swedish sample and were less positive to SA than counselors in the US sample, which represented a larger and more inclusive group consisting of counselors and to some degree social workers. Further, compared to the US sample, the Swedish sample includes more psychologists, who we found had less positive attitudes to SA. Finally, clinicians in the private sector were not included in the Swedish sample; a group with less positive attitudes according to the US study. In addition, the Swedish sample included fewer clinicians with research training (PhD) than the US study, which found high educational level to be a predictor of positive attitudes towards SA.

In this study, we were unfortunately not able to explore the relationship between attitudes and use of SA, which is a limitation. Participants did answer an open-ended question about this but the responses could not be grouped or categorized reliably enough to be included in the analyses.

Finally, the psychometric properties of the subscales, *Utility of Diagnosis* and *Practicality* must be mentioned. In total, all characteristics together explained only 1.8% of the variance in *Utility of Diagnosis*, which is a negligible effect size and less than all other scales. This subscale was also the one with lowest internal consistency and also the one with troublesome face validity in the translation process. The subscale *Practicality* had also questionable internal consistency in the Swedish sample (α = .60) lower than in the US study (α = .75) [26]. In the present study, we chose not to change the number of items to improve the reliabilty since we wanted to compare the results with those in the previous study.

### Implications and conclusion

This study aims to investigate clinician´s attitudes towards standardized assessments and usefulness of diagnosis. The overall positive attitudes toward diagnosis and SA are of importance in the development of EBA within child and adolescent mental health services and our study suggest that clinicians in general value diagnosis and are willing to use SA. When implementing new methods in practice, there are general as well as specific resistances that needs to be overcome and studies in different cultural settings are of importance to further extend the knowledge of what is universal and what is contextual. Our study indicates that there are some differences compared to earlier studies that could be explained by cultural circumstances and may be used for assisting favorable EBA progress in several settings. Nevertheless, there are specific issues that need to be addressed in order to achieve an equitable and efficient health care e.g. the lack of translated assessment tools and training. The health services and the scientific community need to collaborate to succeed in implementing more evidence-based assessments of mental disorders in children and adolescents.

## Abbreviations

CAMHS Stockholm: Specialist Child and Adolescent Mental Healths Services within Stockholm County Council
EBM: evidence-based medicine
EBP: evidence-based practice
EBT: evidence-based treatment
EBA: evidence-based assessment
SA: standardized assessments
